# Crevasse refreezing and signatures of retreat observed at Kamb Ice Stream grounding zone

**DOI:** 10.1038/s41561-023-01129-y

**Published:** 2023-03-02

**Authors:** J. D. Lawrence, P. M. Washam, C. Stevens, C. Hulbe, H. J. Horgan, G. Dunbar, T. Calkin, C. Stewart, N. Robinson, A. D. Mullen, M. R. Meister, B. C. Hurwitz, E. Quartini, D. J. G. Dichek, A. Spears, B. E. Schmidt

**Affiliations:** 1 School of Earth and Atmospheric Sciences, Georgia Institute of Technology, Atlanta, GA USA; 2 Department of Earth and Atmospheric Sciences, Cornell University, Ithaca, NY USA; 3 Honeybee Robotics, Exploration Systems, Altadena, CA USA; 4 Department of Astronomy, Cornell University, Ithaca, NY USA; 5 Ocean Dynamics Group, National Institute of Water and Atmospheric Research (NIWA), Wellington, New Zealand; 6 Department of Physics, University of Auckland, Auckland, New Zealand; 7 School of Surveying, University of Otago, Dunedin, New Zealand; 8 Antarctic Research Centre, Victoria University of Wellington, Wellington, New Zealand

**Keywords:** Cryospheric science, Physical oceanography, Climate sciences

## Abstract

Ice streams flowing into Ross Ice Shelf are presently responsible for around 10% of the mass flux from West Antarctica, with the noteworthy exception of Kamb Ice Stream, which stagnated in the late 1800s. The subsequent reduction in ice supply led to grounding-line retreat at the coastal margin where Kamb transitions into the floating Ross Ice Shelf. Grounding-line migration is linked to broader changes in ice-sheet mass balance and sea level, but our understanding of related ice, ocean and seafloor interactions is limited by the difficulty in accessing these remote regions. Here we report in situ observations from an underwater vehicle deployed at Kamb that show how fine-scale variability in ice and ocean structure combine to influence a diversity of ice–ocean interactions. We found a stratified water column within a tenth of a degree of freezing at the ice base and mapped basal crevasses with supercooled water and active marine ice formation. At the seafloor, we interpret parallel ridges as crevasse impressions left as the ice lifted off during grounding-line retreat. These observations from a recently ungrounded sub-shelf environment illuminate both the geomorphological signatures of past grounding-line retreat and the fine-scale sensitivity of ongoing ice–ocean interactions to ice topography.

## Abstract (web summary)

Observations from a remotely operated underwater vehicle reveal crevasse refreezing and the fine-scale variability in ice and ocean structure at the Kamb Ice Stream grounding line in West Antarctica.

## Main

Along Antarctica’s coast, floating ice shelves buttress the grounding-line (GL) positions of their tributary ice streams and glaciers^1–3^ and influence Southern Ocean circulation^4^. GLs are dynamic transition zones where ice sheets flow into ice shelves, and GL positions reflect a balance of glacial, marine and geologic factors. Changes in GL position are linked to broader changes in ice-sheet mass balance^1,2,5^; for example, ice-shelf thinning due to reduced ice flux can cause GL retreat^6,7^, or alternatively, increased ocean melting can cause ice-shelf thinning, GL retreat and accelerated ice flow that compound ice-sheet mass loss^8–10^.

Earth’s largest ice shelf by area is the Ross Ice Shelf (RIS), which represents a third of all floating glacial ice^3^. The West Antarctica ice streams that feed RIS flow at speeds of hundreds of metres per year but are known to cyclically slow, stagnate and reactivate on century timescales^11,12^. Of these, Kamb Ice Stream (KIS) has been stagnant since ~1865^13–15^. The shift from flow speeds of 350 m yr^−1^ in the main KIS trunk^16^ to now near-zero surface velocities at the KIS GL^17^ is thought to be due to a change in the subglacial water system^13,14,18^. Stagnation initiated an ongoing accumulation of ice upstream^19^ that presently offsets 25% of the mass loss from West Antarctica^20^. Reactivation^18,21,22^ could increase Antarctica’s current contribution to sea-level rise^23^ by 12% (ref. ^24^). Along the KIS coast, reduced ice flux post-stagnation also led to thinning and GL retreat until at least the 1980s^6,7,15^, but a lack of in situ observations from remote GL environments limits our understanding of ice, ocean and seafloor interactions as GLs migrate during cycles of ice-stream activity.

**Extended Data Fig. 1:**
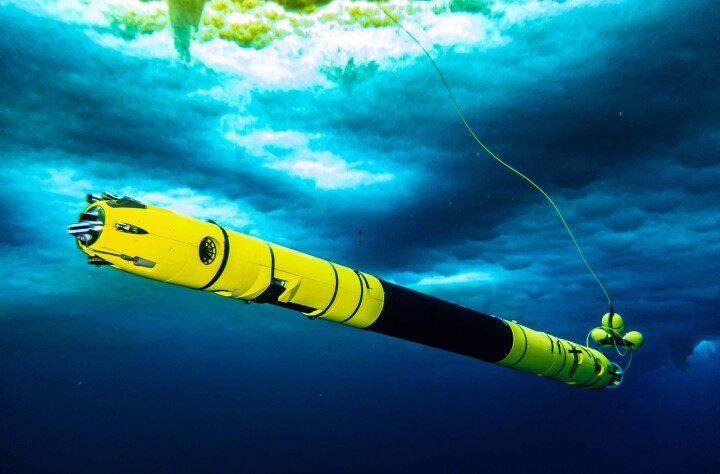
An Icefin ROV below sea ice in McMurdo Sound, near the Ross Ice Shelf terminus in November 2019. The vehicle deployed at KIS1 was configured identically except with optical and electrochemical sensors (Extended Data Table 1) in the forward instrument bay instead of the bathymetric mapping sonar equipped here. Photo courtesy of Rob Robbins, US Antarctic Program. Source data

**Extended Data Fig. 2:**
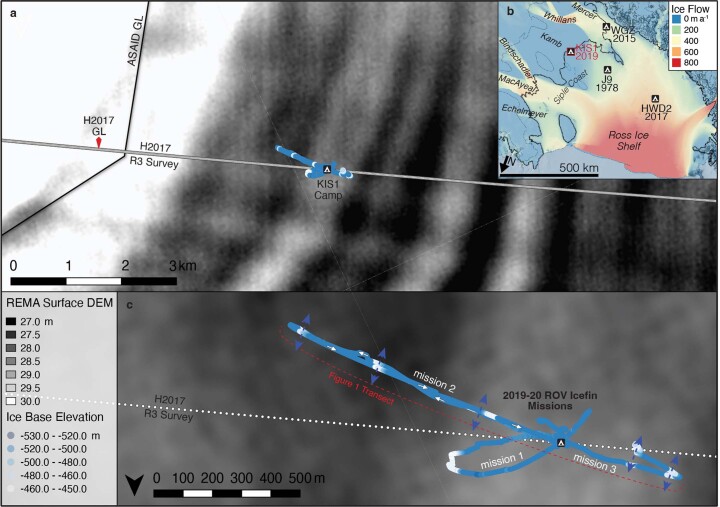
Plan view of the three ROV Icefin missions and orientation relative to prior surveys and the approximate grounding line. **a**,**b**, Inset map (**b**) ice velocities are from MEaSUREs^64^, and the upper panel (**a**) corresponds to the area below the KIS1 2019 camp icon. **c**, A further zoom in on the ROV Icefin survey track (blue shaded circles), which is colored by ice base elevation to highlight crevasse locations. White dotted line indicates the R3 survey from Horgan, et al. (H2017)^6^. Red dotted line indicates the composite transect shown in Fig. 1, and blue dotted arrows show the location and approximate orientation of basal crevasses (elevation data are mismatched for outbound and return tracks due to accumulated position error; see Methods). Surface elevations are sourced from the Reference Elevation Model of Antarctica (REMA)^65^, black grounding line in **a** is from ASAID^66^, and all maps were prepared using QGIS^54^ with the Quantarctica package (V3)^55^. Source data

To survey contemporary processes and infer the post-stagnation sequence at the KIS GL, we deployed the remotely operated underwater vehicle (ROV) Icefin^25,26^ (Extended Data Fig. 1 and instrumentation in Extended Data Table 1) into the 30-m-tall ocean cavity 4.2 km seaward of the contemporary GL^6^ (KIS1 site: 82.7841° S, 155.2627° W), in conjunction with Aotearoa/New Zealand’s Ross Ice Shelf Programme and Antarctic Science Platform. We completed three missions (Extended Data Table 2) for a cumulative 5 km survey (Extended Data Fig. 2) below the 580-m-thick ice.

## Under-ice features and seafloor bedforms

**Fig. 1:**
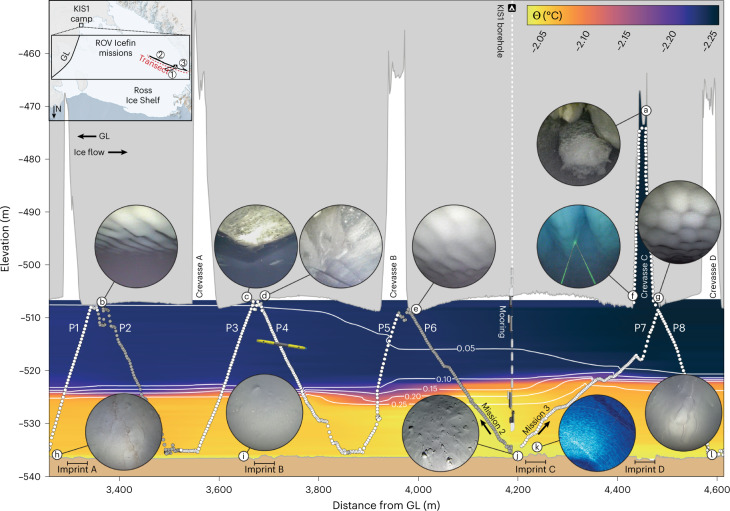
Integrated ROV observations illuminate ice, ocean and seafloor connections in a recently ungrounded region along the KIS coast. The background contour shows Conservative Temperature (Θ), with white contour lines indicating temperature difference from the freezing point (Θ – Θ_fp_). Imagery insets (Supplementary Videos 1–4 and Extended Data Fig. 4) are as follows: **a**, 1.30-m-wide marine ice formations; **b**,**e**, ~0.65-m-wavelength, ~5-cm-amplitude ripples in meteoric ice; **c**, centimetre-scale grain boundaries in basal ice; **d**, layers of sediment-laden basal ice; **f**, 3.50 -m-wide incised runnels; **g**, 0.55- to 0.60-m-wide dimpled meteoric ice; **h**,**l**, ~0.10-m-wavelength seafloor ripples; **i**, smooth seafloor below basal ice; **j**, centimetre-scale clasts; **k**, sonar image of a 20-m-wide crevasse imprint. This transect is generated from the outbound legs of the second (upstream) and third (downstream) missions, which occurred 63 hours apart at reciprocal headings (see map inset and Extended Data Fig. 2). Data are interpolated between white points (the vehicle track), and grey points indicate ocean data filtered out due to aberrant conductivity readings. Elevation is metres below local sea level; distance is reported relative to the previously mapped GL position^6^. P1–P8 correspond to sections plotted as profiles in Fig. 2; 10× vertical exaggeration. Source data

**Fig. 2:**
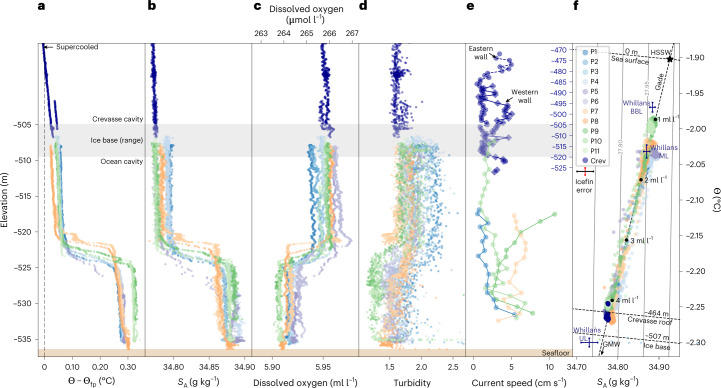
The water column at KIS GL is stratified with a colder and fresher upper layer above a warmer, more-saline lower layer. **a**–**e**, Vertical profiles of water-column properties; P1–P8 correspond to Fig. 1, and P9–P11 correspond to three profiles from the first mission (Fig. 1 insets and Extended Data Fig. 2). Data collected within crevasses (Crev) are displayed in dark blue, and (in **a**–**d**) on a vertically compressed secondary *y* axis. Thermal driving (Θ – Θ_fp_) (**a**) shows waters adjacent to the ice base are within 0.1 °C of Θ_fp_; and waters near a crevasse ceiling are colder than the Θ_fp_ (supercooled). Salinity (*S*_A_ (g kg^−1^)) (**b**) shows an ~0.1 g kg^−1^ halocline separating two well-mixed layers, with the fresher upper layer also extending into crevasses. Both dissolved oxygen (**c**) and turbidity (**d**) were elevated in the upper layer. Current speeds (**e**) (averaged into 1 m bins) were typically <10 cm s^−1^, although we note not all depths or tidal phases were equitably sampled during the three Icefin missions. **f**, Θ*–S*_A_ diagram with *σ*^Θ^ (potential density anomaly) contours and Gade line^28,29^ (equation (1)). Mean properties of the upper layer (UL, −657.3 m), mixed layer (ML, −661.3 m) and bottom boundary layer (BBL, −665.7 m) observed south of the KIS GL at the Whillans Ice Stream GL^33^ are indicated by blue crosses. Source data

We mapped a variety of related under-ice features and seafloor bedforms with Icefin (Fig. 1 and Supplementary Videos 1–4). Most prominent were five 45- to 55-m–tall and wide basal crevasses, with long axes aligned orthogonal to the ice flow. The ice base between crevasses was concave, potentially indicating viscous deformation^27^ that evolved post-flotation. Upstream (eastern) crevasse walls were more steeply sloped than downstream walls, and asymmetric water circulation was measured within a crevasse cavity (Fig. 2). Marine ice (refrozen seawater) was observed along the upper sidewalls and ceiling of Crevasse C (Fig. 1a).

The borehole sidewall and most of the ice-shelf base were composed of cloudy meteoric ice (Fig. 1b,e-g). However, approximately 500 m upstream of the borehole, we also observed a 200-m-wide section of laminated, sediment-laden ice (Fig. 1c,d). We interpret this as ‘basal ice’, formed as turbid subglacial freshwater froze to the ice base before ungrounding^21,22^. All of the level ice-shelf base was rippled (Fig. 1b–e), with a short axis wavelength of 0.65 ± 0.10 m.

## Water-column properties

The water column was stratified with two well-mixed layers of similar thicknesses (Fig. 2) separated by a 2- to 3-m-thick pycnocline, and both layers were colder than the surface freezing point. Thermal driving, the difference between the water temperature and in situ (pressure-depressed) freezing point (Θ – Θ_fp_), ranged from +0.02 to +0.08 °C in the upper layer and from +0.25 to +0.34 °C in the lower layer (Fig. 2a). Current speeds ranged from 2 to 8 cm s^−1^ and peaked at 13 cm s^−1^ (Fig. 2e). From upper-layer water properties, current speeds and a drag coefficient constrained by sonar measurements of ice topography (Supplementary Information), we estimate the local ice-shelf melt rate was 0.26 m yr^−1^.

The linear trend in Θ and *S*_A_ (Fig. 2f) results from mixing between two water masses^28,29^, high-salinity shelf water (HSSW) and fresh glacial meltwater (GMW). Dense HSSW is generated in the Ross Sea polynyas as brine is rejected during sea-ice formation, and a portion subsequently advects below the RIS, where it drives melting at the GL^4,30,31^. Resultant production of GMW at the colder, pressure-depressed freezing point (which decreases ~0.1 °C per 130 dbar) freshens and cools the HSSW. The stratification between the two layers reflects different GMW concentrations, which are quantified by their position along the Gade mixing line^28,29^ (equation (1)), where the upper and lower layers contained GMW concentrations of 4 ml l^−1^ and 1 ml l^−1^, respectively (Fig. 2f). Dissolved oxygen concentrations are elevated in the upper layer as melting meteoric ice releases trapped bubbles of atmospheric gas^29,32^ (Fig. 2c), and elevated upper-layer turbidity is (Fig. 2d) is attributed to sediment sourced from melting basal ice (Fig. 1c,d) as no turbid subglacial outflow was detected.

The only previous oceanographic measurements along the 800 km Siple Coast were recorded in a stratified 10 m water column at the Whillans Ice Stream GL (Extended Data Fig. 1b) in 2015^33^. Maximum upper-layer thermal forcing was similar to that of KIS at +0.1 °C (UL; Fig. 2f). The underlying mixed-layer (ML; Fig. 2f) temperature and salinity at Whillans match those of the lower layer at KIS1. However, a warmer, more-saline bottom boundary layer (BBL; Fig. 2f) observed at Whillans more closely resembled pure HSSW than did the majority of lower-layer profiles we collected. The water-column structure at KIS shows that while HSSW reaches the Siple Coast RIS GL, the degree to which it is modified en route by melting and GMW input depends on ice-shelf draft and seafloor bathymetry^31,33–35^.

## Refreezing in a basal crevasse

**Fig. 3:**
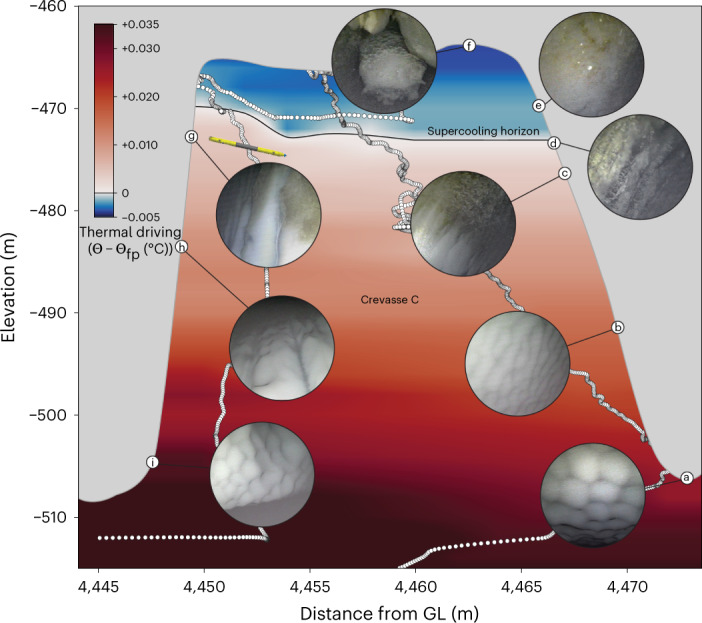
Supercooled water and refreezing in a basal crevasse. Background thermal driving contour in the crevasse water column (Supplementary Video 4 Extended Data Fig. 5) is interpolated from white sample points (vehicle track), with a 0 °C contour line demarking the supercooling horizon. Insets **a**–**i** illustrate the different ice textures and morphologies in order of encounter (full images in Extended Data Fig. 5); **c** and **g** show the onset of marine ice along opposite crevasse sidewalls at nearly the same height. The vehicle path is misaligned by ~5–10 m from the ice profile due to motion south along the long axis of the crevasse (into the page) and accumulated vehicle positioning error (Supplementary Information); however, all data (and the length of ROV Icefin) are to scale; 2× horizontal exaggeration. Source data

We found supercooled water and marine ice formation along the upper sidewalls and ceiling of Crevasse C (Fig. 3 and Supplementary Video 4). Below an ice shelf, supercooling occurs when seawater cooled by GMW upwells such that the pressure reduction raises the freezing point above the water temperature^4^. As Icefin navigated up the downstream wall of Crevasse C, thermal driving steadily decreased, and at −475.4 m, the ice texture and colour shifted abruptly from smooth, melting, cloudy meteoric ice (Fig. 3a,b) to green-tinged ice with incised runnels tens of centimetres wide (Fig. 3c). At −473.4 m, thermal driving became negative (supercooled), and the ice appeared rougher with centimetre-scale green-tinged crystals (Fig. 3d), which we interpret as the onset of active ice accretion.

Continuing upwards to the top of the crevasse, the ice we observed transitioned through increasingly green and crystalline facies (Fig. 3e). Thermal driving reached a minimum of −0.006 °C in the crevasse ceiling, where we found a 2- to 3-m-wide recessed trench filled with rounded, globular formations tens of centimetres across with linear channels and brinicle-like protrusions (Fig. 3f). This process of melting, upwelling and refreezing driven by topography in submerged ice is referred to as an ice pump^30,36^, and it primes seawater for refreezing and marine ice formation on regional scales as well^37,38^. Marine ice growth through ice pumping is an important process that can act to mechanically stabilize basal crevasses and rift zones^27,39^ and is the origin of green, or ‘jade’, icebergs^38,40^.

Along the steeper upstream wall, we observed a similar metre-wide transition band from accretion ice back to meteoric at −471.5 m (Fig. 3g), with larger vertical runnels that deepened and widened farther down the wall to ~2 m incisions with 4 m wavelengths. Runnels may be incised as brine rejected from accreting ice runs downward along the wall and melts ice below. We rule out formation driven by upwelling water^41^ as runnels were present above the supercooling horizon and did not extend to the lower crevasse edge. Greenish debris accumulated on centimetre-scale meteoric ice cusps observed within runnel apexes may be organics or mineral precipitates^40^ released from re-melting accretion ice. This is consistent with the presence and ablated appearance of the lower accretion band just below the contemporary supercooling horizon and actively forming ice (Fig. 3c,g).

## Seafloor bedforms

**Fig. 4:**
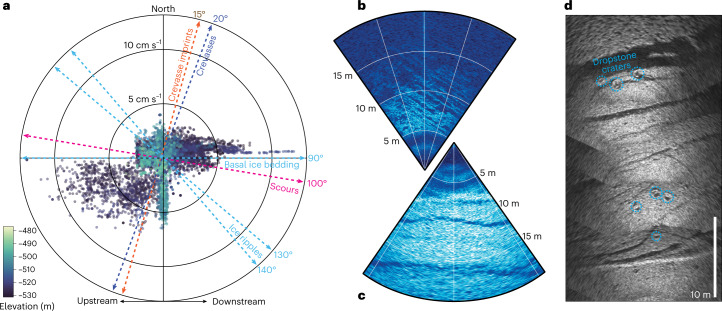
Relative feature orientations link seafloor, ocean and glacial processes. **a**, Polar plot relating feature orientations (in degrees, relative to true north) in the Kamb GL ocean cavity with current velocities (in cm s^−1^), coloured by elevation. In the crevasse, there was strong southward flow at the western (downstream) wall and weaker northward flow at the eastern (upstream) wall. Crevasses and crevasse imprints were both orthogonal to ice-flow direction (westward). Ice-base ripples were orthogonal to mean along-shore current (southwest–northeast) flow. **b**, Sonar observations of seafloor scours. **c**, Sonar image of crevasse imprint B (Fig. 1). **d**, Sonar mosaics of crevasse imprint A (Fig. 1), with dropstone craters. Source data

At the seafloor, we observed four stratigraphically superposed bedforms (Fig. 4 and Supplementary Videos 1 and 2): scours (Fig. 4b), crevasse imprints (Figs. 1 and 4c,d), dropstone craters (Fig. 4d) and meandering surface ripples (Fig. 1h,l). The lowermost bedform consisted of parallel striations tens of metres long aligned to the ice-flow direction, which we interpret as glacial scours, carved by clasts embedded in basal ice before KIS stagnation and GL retreat. The ridges were ~0.1 m in height and ~0.1–0.5 m wide (Fig. 4b) and most apparent in orthogonal sonar imagery, which penetrated the thin sediment drape visible in optical imagery (Supplementary Video 1). Scours are nearly ubiquitous and morphologically similar to fluting found below Mackay Glacier in the Ross Sea^41^ but are smaller amplitude than previously observed sub-ice-shelf keel scours^42–44^.

Scours were cross-cut by four parallel sets of linear ridges (Figs. 1 and 4c,d) oriented across the ice-flow direction at strike angles of ~15° (Fig. 4a). The ridges were 20–40 m wide and at least 100s of metres long with 0.5–1.0 m relief from depressed margins to central plateaus. The strike, width and length of the ridge sets parallel the contemporary basal crevasses, and we interpret them as crevasse squeeze ridges formed when the GL was nearby during post-stagnation retreat^42,45,46^. The correspondingly uneven spacings (within navigational uncertainty; Extended Data Table 2) of both basal crevasses and crevasse imprints suggest that the crevasses we observed were responsible for imprint formation. Similar features were observed at Mackay Glacier where soft sediment was shaped as ice at the GL rose and fell with the tide^41^.

Cobble- and boulder-sized dropstones with craters up to 0.50 m in diameter (Fig. 4d) were evident throughout the survey, indicating that an expansive layer of accreted basal ice has since melted back. The crevasse imprints below meteoric ice (Figs. 1 and 4d) and basal ice (Figs. 1 and 4c) both exhibit dropstone craters, which supports imprint formation at the onset of ocean cavity formation.

**Extended Data Fig. 3:**
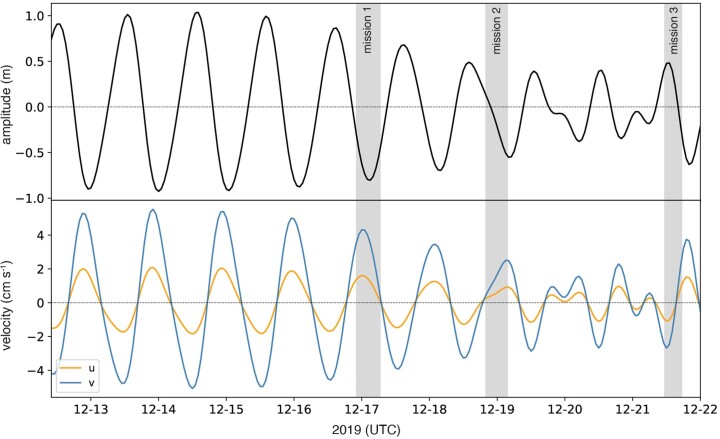
ROV Icefin mission timing, relative to modeled tidal amplitude and currents. (CATS2008;^67^ at −82.78°, −155.26°). Source data

**Extended Data Fig. 6:**
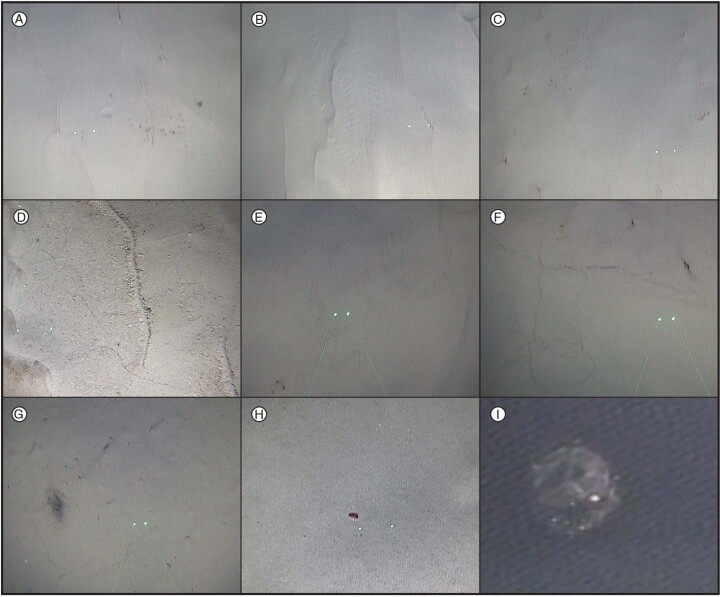
Evidence of macrofauna observed with ROV Icefin near the Kamb Ice Stream Grounding Line. The lack of sessile macrofauna supports recent seafloor exposure from grounding line retreat and ongoing sediment deposition, but there are several features indicating the presence of small animals via tracks and other evidence of bioturbation. Laser scale is 5 cm where present, and the object in panel i is estimated at <2 cm across. Please contact the author if interested in this imagery.

Centimetre-scale ripple bedforms cover much of the seafloor, including dropstone craters, but are not present below the patch of basal ice. There the seafloor was smoother, with unmodified craters and unburied clasts indicative of continued debris delivery (Fig. 1i). Ripple morphology suggests formation in 20 cm s^−1^ currents^47^. However, contemporary measurements from Icefin (Fig. 3a) and tidal model predictions (Extended Data Fig. 3) are below this threshold. Together with their absence below basal ice (where sediment deposition continues), this indicates that the ripples formed during or shortly after ice lift-off in a thin, tidally modulated water column and are no longer active. Other centimetre-scale seafloor features include morphological indications of macrofauna (Extended Data Fig. 6).

At the KIS1 borehole, a 0.50-m-deep gravity core recovered poorly sorted debris typical of basal ice sediment (diamicton)^42,47^, and previous drilling surveys through the KIS trunk 275 km upstream found 15 m of basal ice bearing the equivalent of ~2 m of sediment^21,48^. Together with indications of a formerly expansive basal ice layer across this transect, post-stagnation sediment deposition might have been expected to completely bury inactive bedforms of lesser amplitude—including scours, centimetre-scale ripples and crevasse impressions. That these features remain visible throughout much of our survey supports spatial variability in sediment deposition from melting basal ice, in turn indicative of sub-kilometre-scale variability in basal ice-accretion rates below grounded ice^14^.

## Integrated sub-shelf processes

We used an ROV to map ice, ocean and seafloor interactions in the recently formed ocean cavity seaward of the KIS GL^6,49^. The 30 m water column was separated into two layers, with a colder upper layer that limited heat flux from the warmer lower layer to the ice base throughout this survey, within ~3.30 km of the contemporary GL. We mapped multiple crevasses in the ice-shelf base and observed supercooled water and marine ice refreezing within a crevasse. Crevasse distribution and refreezing processes are linked to overall ice-shelf mechanical strength^27,39^ but are difficult to observe with remote-sensing techniques. These in situ observations demonstrate the sensitivity of ice–ocean interactions to decametre-scale topography and temperature variability of thousandths of a degree.

At the seafloor, impressions left by basal crevasses as the ice lifted off support post-stagnation GL retreat^6,15,46^ and establish a relationship that may inform interpretation of the geomorphological record in other deglaciating regions^42^. Previous work at KIS measured GL retreat at 28 ± 5 m yr^−1^ in the decade following 1974^49^, and the modern satellite record shows that the GL has been static at the contemporary location ~4.2 km east of the KIS1 site since then^6,15,17,50^. Combined with the relatively lower ice-shelf melt rates of 0.26 m yr^−1^ (compared with, for example, tens of metres per year melt rates at some Amundsen Sea glaciers^3,8^) estimated here, this evidence reinforces the role of grounded ice-stream dynamics in driving the variability of cold-cavity^3^ ice-shelf GLs^6,7^.

The survey reported here contributes a unified perspective on extant and past GL processes and demonstrates sub-kilometre-scale environmental variability below ice. This motivates continued efforts to pair the spatial context provided by ROV systems with traditional profiling instruments and long-duration mooring installations, which together can provide a complementary spatial and temporal understanding of the environments and processes below ice shelves.

## Methods

### ROV Icefin specifications

The ROV Icefin (Extended Data Fig. 1), developed in Dr. Britney Schmidt’s Planetary Habitability and Technology Lab, is designed for sub-ice oceanography and surveying and can be deployed through sea-ice or ice-shelf boreholes of ≥35 cm diameter. At KIS1, Icefin was configured (Extended Data Table 1) to measure conductivity, temperature, pressure, dissolved oxygen, pH, redox potential, chlorophyll *a*, turbidity, dissolved organics and current speeds. For optical imaging, Icefin had two standard-definition forward cameras, one standard-definition aft camera and a 1,080p (progressive-scan) downward-looking camera with 5 cm scaling lasers. For acoustic imaging and sounding, Icefin had a forward imaging multibeam, upward-looking single-beam altimeter and a downward-looking Doppler velocity logger (DVL) for navigation with acoustic Doppler current profiling and altimetry. Real-time telemetry and data were relayed topside during missions through a Kevlar-reinforced 4.3 mm neutrally buoyant fibre-optic tether (Linden Photonics SPE7094) and recorded using Greensea Systems Balefire software with Lightweight Communications and Marshalling for data handling^51^. Uncompressed video was captured at 30 frames per second.

### Underwater navigation

Vehicle navigation and data georeferencing are accomplished by dead reckoning with the DVL, which determines *x*, *y* and *z* vehicle velocities from the Doppler shift of four independent sonar beams reflecting off the seafloor. These velocities yield speed and course over ground in the vehicle frame, which are combined with a heading held by an inertial measurement unit to orient velocities in the Earth frame. Each sample is integrated from the initial Global Positioning System (GPS)-derived latitude and longitude^52^ at the surface to generate a mission track. A navigational challenge at high latitudes with near-vertical magnetic-field inclinations and high declinations is accurately determining the initial vehicle heading^53^. For this work, we employed a method by which heading was found on the surface as the bearing between two GPS-derived points aligned to the vehicle, using a long baseline (≥50 m) to reduce error to ~1°.

Navigational error accumulates in the position solution over the duration of a mission, and maximum error can be estimated as the distance between the borehole location in software and the actual borehole location the vehicle returns to. This distance was typically <50 m for missions up to 1 km in range (Extended Data Table 2). The primary source of error is lost DVL ‘lock’, when the DVL does not receive sufficiently strong echoes from at least three of the four beams and cannot calculate vehicle velocities for that interval. Lock is typically regained in <1 s over flat interfaces, unless the vehicle is executing a high-pitch manoeuvre or DVL lock has been intentionally de-prioritized to enable an observational priority (such as the operation at high-pitch angles during the crevasse survey). All Icefin missions, basemaps and geospatial data are collated using the Quantarctica package for QGIS v.3^54,55^.

### Vehicle operations

KIS1 Camp (82.7841° S, 155.2627° W) was established in November 2019. The Antarctica New Zealand team began drilling at HWD-1 on 11 December and completed a 22-cm-diameter pilot hole on 14 December. Ice-shelf thickness was 583.4 m, with a density-corrected surface elevation (correction described in Reference datums) of 76.9 m. Later reaming widened the borehole to at least 35 cm to enable ROV Icefin deployment. The hole was maintained by regular reaming until 23 December. As one component of the broader science programme, Icefin completed three missions under ice between 17 and 21 December (Extended Data Fig. 3 and Extended Data Table 2).

After transiting the borehole, before each mission began, we thermally equilibrated the vehicle at the ice-shelf base^56^ and then completed a vertical cast to the seafloor before dropping a plastic-free ballast weight to reorient the vehicle from a vertical to horizontal attitude. To balance water-column profiling and long-distance survey priorities, during outbound transects, we flew a sawtooth pattern in the 30 m cavity (Fig. 1). During climbs, we held approximately +25° pitch utilizing Icefin’s two Z thrusters, at forward speed of 0.2–0.3 m s^−1^. Icefin was ballasted negative by a few tens of grams, enabling descents at roughly level pitch and similar forward speeds. Sawtooth profile wavelength was 250–400 m, and we held altitude at the seafloor or ice interface for 30–50 m of visual inspection at ≤2 m ranges. After reaching the maximum planned range for a mission, observational priorities shifted to visual and acoustic survey, and the vehicle was ‘reeled in’ by the tether while using thrusters to hold level pitch and altitude of 1–3 m over the seafloor. This strategy maximized battery life, improved the quality of ice and seafloor altimetry measurements and permitted continuous video survey of the seafloor for later mosaic and structure from motion reconstructions^47^.

Of note for future borehole work in cold-cavity ice shelves, we observed localized frazil ice accumulation in a narrow metre-wide band below the borehole at the end of a mission. We attribute this to freezing of brackish water displaced by warm water pumped into the borehole to unfreeze the sea surface at the beginning of one of the missions. This localized ice growth was observed in forward sonar and optical imagery. Freezing in general is also a challenge during vehicle recovery through the borehole as the brackish water on the vehicle freezes rapidly in the −25 °C air that blows through the porous firn above freeboard. As such, instrumentation was selected to be as robust to freezing as possible (for example, physical or optical sensors, externally flushed CT cell with no internal pumps or plumbing prone to ice-induced failure). This permits Icefin to operate in supercooled waters and to be stored ‘on ice’ outdoors between missions, which greatly eased operational complexity and impact to the larger scientific programme.

### Reference datums

Elevations (m) are reported with respect to local sea level, where the sea surface is 0 dbar (sea pressure), and are converted using the Gibbs Seawater (GSW)^57,58^ Toolbox z_from_p function considering sample latitude. Correcting measured freeboard (65 m) in the freshened borehole water column to mean seawater density at that depth (1,027.95 kg m^−3^, from pre-field deployment conductivity, temperature and depth (CTD) casts completed in McMurdo Sound) yields a freeboard, or surface elevation, of 77 m. The mean World Geodetic System 1984 ellipsoid surface elevation at camp was 27.5 m, so we note an ellipsoid–geoid offset of approximately −49.5 m at the KIS1 site. While ROV navigation underwater is referenced to sea pressure (0 dbar at sea surface, with local atmospheric pressure subtracted), this offset is noted for comparison with data referenced to ellipsoid elevation. Horizontal distances are indexed to GL position by compressing the 1.5 km composite section (Fig. 1) to the GL mapped in previous radar and seismic surveys (Extended Data Fig. 2, R3 Survey^6^).

### Conductivity and temperature data processing

The NBOSI cabled conductivity and temperature (CT) sensor does not include a pressure sensor, so temperature and conductivity data were processed using the pressure calculated after correcting for the mounting offset to the primary vehicle pressure sensor and vehicle pitch. To obtain sea pressure, atmospheric pressure recorded just above the sea surface before each mission is subtracted. Despite routine calibration, previous fieldwork in McMurdo Sound established a linear temperature and conductivity offsets (biases) between the NBOSI cabled CT sensor when compared with the adjacent dissolved oxygen optode thermistor and an RBR Concerto3, where the latter two sensors consistently agreed. We treat this operationally by conducting an independent CTD cast immediately before or after each Icefin mission and then applying a constant offset based on observed water-mass properties by each sensor. Temperature offsets generally on order of 10–30 mK have persisted through pre- and post-season calibration efforts. Offsets in conductivity space are more difficult to constrain as the NBOSI CT is the only onboard conductivity sensor, but through co-calibration at KIS1 with two RBR Concerto3 profiling CTDs (C. Stevens, personal communication), we observe it to range between +0.01 and 0.25 mS cm^–1^. For these missions, the applied offsets were +0.0175 °C and +0.249 mS cm^–1^.

Conductivity values are further quality controlled for low readings resulting from low vehicle velocities (causing poor CT flushing) or the passage of ice or sediment particulate, which reduces the bulk conductivity of the sensing volume. Corrected and filtered data were then processed according to the Thermodynamic Equation of Seawater 2010^57^ using the Python3 implementation of the GSW Toolbox^58^ to derive *S*_A_, Θ, the potential density anomaly (*σ*^0^) and finally thermal driving. Contour plots and vertical profiles are generated by down sampling and averaging observations to 1 Hz, followed by applying a 4 s rolling mean and linear interpolation across a regularly spaced grid. Oceanographic visualizations follow best practices described in ref. ^59^.

Mixing in the KIS water column falls along a Gade line calculated using HSSW *S*_A_ and Θ endmember properties, following Wåhlin et al.^60^, after Gade^28^ (equation (1); see also ref. ^29^).

1 $$T_{\mathrm{P}}\left( {S_{\mathrm{A}}} \right) = T_{{\mathrm{ocean}}}\frac{{L_{\mathrm{F}}}}{{c_{\mathrm{p}}}}\left( {1 - \frac{{S_{{\mathrm{ocean}}}}}{{S_{{{\mathrm{A}}}}}}} \right)$$

The terms *T*_ocean_ and *S*_ocean_ are HSSW temperature (−1.903 °C) and salinity (34.925 g kg^−1^), respectively, *L*_F_ is the latent heat of fusion for ice and *C*_P_ is the specific heat capacity of water at HSSW temperature, salinity and pressure. These properties are taken from a range of HSSW observations made at similar depths during past Icefin missions in McMurdo Sound (near the RIS terminus and polynya). Fresh GMW concentrations of the KIS water-column layers are calculated from the slope of the Gade line.

### Current-speed processing

Ocean current speeds come from the LinkQuest NavQuest 600 Micro DVL on board Icefin, which doubles as an acoustic Doppler current profiler (ADCP). The DVL function calculates the *X*, *Y* and *Z* velocities of the vehicle (Icefin) by pinging a static surface, where *X*, *Y* and *Z* are the respective major, minor and vertical axes of the vehicle. As the DVL calculates the vehicle’s velocities, it also retrieves water-column velocities in 2 m bins at a variable start distance from the vehicle. The minimum nominal altitude from an interface for current profiling to occur is 10 m. Gradients in vehicle pitch, roll, heading and speed dictate the distance of the first bin from the vehicle and the sampling frequency, which can reach a maximum of 5 Hz although we subsample velocities to a fixed rate of 1 Hz. The manufacturer-stated accuracies of ADCP current velocities are 1% of the vehicle’s velocity in that direction. As Icefin travels at speeds of ≤50 cm s^−1^, typically in the *x* direction, the uncertainty in vehicle *X* velocity recorded by the ADCP is ≤5 mm s^−1^. Water-column *X* velocities are differenced from the vehicle velocity, resulting in an uncertainty of ≤1 cm s^−1^. Vehicle velocities in the *Y* and *Z* directions are typically much lower than in the *X* direction, so associated water-column uncertainties are likewise lower.

Only *X* and *Y* velocities are analysed in this work. The following post-processing steps were independently applied to each velocity component:

1. Remove measurements if bin depth is below seafloor depth.
2. Remove raw measurements of NaN or exactly 0 m s^−1^.
3. Remove measurements if absolute value of vehicle pitch or roll is greater than 30°.
4. Convert measurements from vehicle reference frame to geographic reference frame.
5. Apply 30 s mean filter.
6. Filter measurements for gradients greater than 1 standard deviation from the mean in vehicle speed, pitch, roll and individual bin velocity.
7. Collate bins by each up and down vehicle swoop into 1 m vertical bins, then remove measurements if they exceed 1 standard deviation of the mean for that 1 m depth range.

After completing these post-processing steps, the *X* and *Y* velocity components were combined into current speeds.

### Altimetry processing

Seafloor elevations are derived from the average range of the four ADCP beams and corrected for tidal variation as recorded by a surface Global Navigation Satellite System station. Overhead ice-ceiling elevations were measured with a single-beam upward-looking altimeter and re-projected to correct for vehicle roll, pitch and yaw with a rotation quaternion.

### Forward multibeam imaging sonar processing

To enable accurate determination of the orientation and scale of ice and seafloor features, ambient ocean properties were input to determine the local speed of sound. Mosaics (Fig. 4) are generated by overlaying and blending sonar frames in post.

### Other processing

Dissolved oxygen data are corrected to compensate for pressure effects and salinity following manufacturer’s procedures ^61^ and recommendations in ref. ^62^, with calibration coefficients determined in ref. ^63^. Seawater oxygen saturation values to derive percentage of saturation for each observation are calculated using the GSW O2sol function ^57,58^. During the third mission, an offset of 8.01 μmol kg^−1^ was applied to an anomalous section of data from 21 December 2019 11:20:46 to 21 December 2019 15:10:30 utc on the basis of values for the same water mass observed during previous missions. The anomalously offset readings may have been due to transient ice formation or interference with the sensing foil.

Raw turbidity, chlorophyll *a* and fluorescent dissolved organic material data are output in counts, which are corrected per the manufacturer’s calibration by the scale value after subtraction of dark (zero) counts. Chlorophyll *a* and dissolved organic material had no systematic variation in the KIS1 water column. The pH and redox signals are output in analogue voltages and calibrated per manufacturer’s procedure. A pH signal, while on the order of sensor resolution, was detectable, and relatively higher, in the upper water layer. Redox voltages had no systematic variation in the KIS1 water column.

## Online content

Any methods, additional references, Nature Portfolio reporting summaries, source data, extended data, supplementary information, acknowledgements, peer review information; details of author contributions and competing interests; and statements of data and code availability are available at 10.1038/s41561-023-01129-y.

### Supplementary information

Supplementary Information Supplementary Sections 1 and 2 and references.

Supplementary Video 1 This video is a clip from a synchronized composite of Mission 2 showing seafloor Imprint A. Forward-looking multibeam sonar, forward-looking upper and lower analogue cameras and high-definition downward-looking camera streams are shown. Overlays (r, p, y) indicate the vehicle attitude in degrees of roll, pitch and yaw. ‘SOG’ indicates speed over ground in m s^−1^, and ‘range’ is the straight-line distance between the vehicle and the borehole. ‘Z_ice’, ‘Z_veh’ and ‘Z_bed’ indicate the elevation of the ice base overhead, vehicle and seafloor, respectively. ‘CT’, ‘SA’, ‘thermal driving’ and ‘DO’ indicate Conservative Temperature (Θ, °C), Absolute Salinity (g kg^−1^), thermal driving (Θ - Θ_fp_) and pressure- and salinity-corrected dissolved oxygen concentrations (µmol l^−1^), respectively. Laser scale is 5 cm when present.

Supplementary Video 2 This video clip depicts basal scours in the seafloor imaged during Mission 2. Layout and overlays are as defined for Supplementary Video 1.

Supplementary Video 3 This video is a clip from Mission 2 showing sediment-laden basal ice at the ice-shelf base. Layout and overlays are as defined for Supplementary Video 1.

Supplementary Video 4 This video is a clip from the crevasse survey at the end of Mission 3. Layout and overlays are as defined for Supplementary Video 1.

### Source data

Source Data Fig. 1 This file contains merged Icefin mission data used to create figures showing ice, ocean and seafloor parameters.

Source Data Fig. 2 This file contains merged Icefin mission data used to create figures showing ice, ocean and seafloor parameters.

Source Data Fig. 3 This file contains merged Icefin mission data used to create figures showing ice, ocean and seafloor parameters.

Source Data Fig. 4 This file contains merged Icefin mission data used to create figures showing ice, ocean and seafloor parameters.

Source Data Extended Data Fig. 1 Extended Data Fig. 1 is a colour-corrected version of this digital image.

Source Data Extended Data Fig. 2 This file contains merged Icefin mission data, including the vehicle path and ice-shelf geometry.

Source Data Extended Data Fig. 3 This file includes the tidal model data for the figure (see citation).

Source Data Extended Data Table 2 Extended Data Table 2 is calculated as described in the caption from aggregate Icefin mission data.

## Figures and Tables

**Extended Data Fig. 4:**
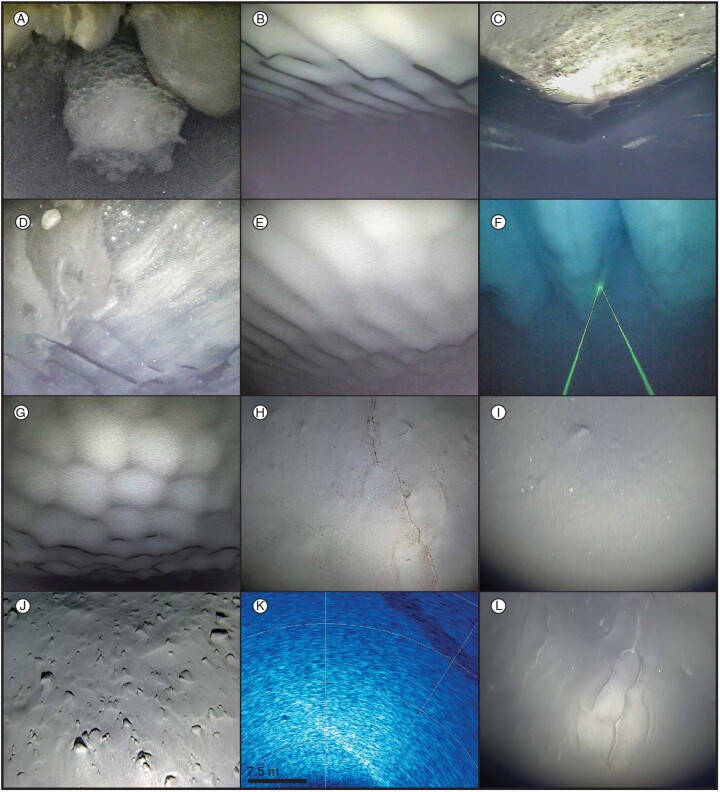
Full images of ice morphologies depicted in Fig. 1. Laser scale is 5 cm where present. See Supplementary Videos 1–4 for synchronized visualization with quantitative sonar scaling.

**Extended Data Fig. 5:**
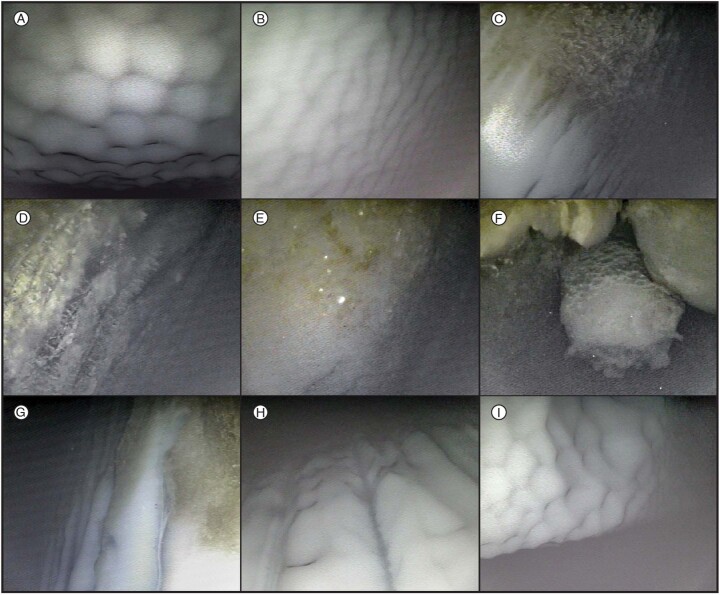
Full images of ice morphologies depicted in Fig. 3. Please view Supplementary Videos 1–4 for synchronized visualization with quantitative sonar scaling.

**Extended Data Table 1:**
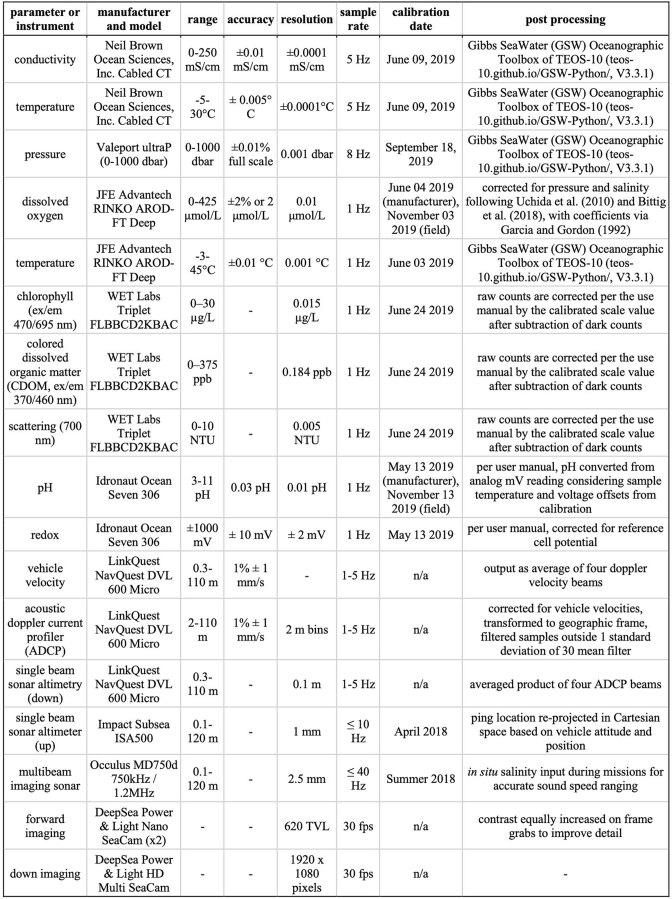
ROV Icefin instrumentation deployed at KIS1

**Extended Data Table 2:**
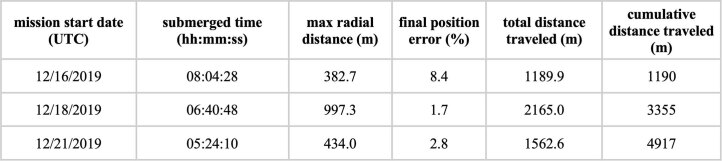
Summary statistics of the three ROV Icefin missions completed at KIS1

Final position error is estimated from the difference between the initial and final vehicle navigation solution at the borehole, as percentage of the overall distance traveled.

Source data

## Data Availability

Data from each Icefin mission at KIS1 are available at https://www.usap-dc.org/. Source data are provided with this paper.

## Code availability

ROV data processing and visualization were conducted in Python, and code is available upon request.
