# Supplementary material for: Crevasse refreezing and signatures of retreat observed at Kamb Ice Stream grounding zone
Source: Nat Geosci. 2023 Mar 2;16(3):238–43. doi: 10.1038/s41561-023-01129-y (PMC10005960; doi:10.1038/s41561-023-01129-y)
Supplement: Supplementary file 1 — Supplementary Sections 1 and 2 and references. [file 41561_2023_1129_MOESM1_ESM.pdf]

nature geoscience

Article

https://doi.org/10.1038/s41561-023-01129-y

Crevasse refreezing and signatures of retreat observed at Kamb Ice Stream grounding zone

In the format provided by the authors and unedited

Supplementary Information

1. Bounding drag from basal ice morphology to estimate melt rates

Improving the fidelity of ice-ocean melt parameterizations is a high priority goal to reduce uncertainty of sea level rise projections[68]. At present, ice shelf melt rates are commonly modeled via a two- or three-equation parameterization suitable for fully turbulent conditions[69]. In the simplified two-equation form[70], which assumes that infinite salt diffusion maintains the basal interface at the marine freezing point, the latent heat loss during melting is equal to the sensible heat flux from the ocean less heat conducted through the shelf. The melt rate a_b can be calculated as

$$a_b = \frac{\rho_w c_w \, u_* \, \Gamma_{TS} \, (T_w - T_F)}{\rho_i (L_i + c_i (T_F - T_i)}$$

$$From\,Thomas, 1979\text{:} \ \lambda = 10^3 \, \nu \, / \, u_* \rightarrow \quad u_* = 10^3 \, \nu \, / \lambda$$

$$yields\text{:} \quad a_b = \frac{\rho_w c_w \, 10^3 \, \nu \, \Gamma_{TS} \, (T_w - T_F)}{\rho_i (L_i + c_i (T_F - T_i) \, \lambda}$$

where $\rho_{w,i}$ is sea water density (observed) and reference ice shelf density (917 kg m^{-3}), L_i is the latent heat of fusion for ice, $c_{w,i}$ is the specific heat capacity of sea water and ice, and T_i is the surface ice temperature of -25°C (observed). T_F, the basal ice temperature assumed at the mixed layer freezing point, is typically solved for with a linearized equation[69]. However, the linear form deviates significantly from the Conservative Temperature freezing point as calculated via a modified Newton-Raphson iteration[71] implemented in The Gibbs SeaWater (GSW) Oceanographic Toolbox of TEOS-10. For cold cavity ice shelves, the disagreement can be of the same order (0.001 °C) of the thermal driving, and so here we use the GSW implementation to calculate T_F.

The product of the two remaining unknowns in Eq. 2, friction velocity u_* and the dimensionless transfer coefficient Γ_{TS}, describe how mixed layer turbulence determines sensible heat transfer to the ice base. The friction velocity is parameterized as $u_* = \sqrt{c_d}\, U$ where U is the ocean mixed layer (far-field) velocity and c_d is a dimensionless drag coefficient that captures the roughness of the basal ice interface[69]. By including a dependence on c_d in u_*, the dependence of Γ_{TS} on roughness is minimized such that a single Γ_{TS} value may be more globally applicable (e.g. 0.006[70]). Presently however, basal roughness is often also held constant in regional melt models, although c_d has been found to vary both seasonally and spatially in polar regions[68]. As such, several studies have sought to bound applicable c_d ranges for typical melting or accreting basal ice morphologies (smooth, rippled, scalloped, or platelet-studded), and have measured friction velocity profiles in the laboratory[72] and below sea ice[68].

To investigate the possibility of a characteristic relationship between friction velocity and ripple wavelength at an interface, Thomas (1979)[73] collated observational and experimental datasets of ripple formation across several fluid and solid interfaces, including ripples on the underside of river ice and in ice exposed to wind. Thomas (1979) found that, for wavelengths (λ) ranging from 5x10^{-5} to 1 m, most published data fit a formula of:

$$\lambda = 10^3\, \nu \,/\, u_*$$

Combining this relationship with $u_* = \sqrt{c_d}\, U$ yields $c_d = (10^3\, \nu \,/\, \lambda\, U)^2$, which we use to solve for c_d from observations of current speed ($U, m\ s^{-1}$) and ice ripple wavelength (λ, m), with the kinematic viscosity of seawater(ν, $1.95\ \times 10^{-6}$ m^{2}s^{-1})[69]. With ROV Icefin's forward-looking multibeam sonar we measured the ice shelf base ripple λ as 0.65±0.1 m, and with the ADCP

function of the DVL observed current speeds of approximately 2 – 13 cm s^{-1}. We were not able to equitably sample U at the ice base across all tidal phases between the three missions, and so estimate $\underline{U}$ at 0.05 m s^{-1}. This yields a c_d value of 3.6×10^{-3}, on order typical values[74] of 3×10^{-3}. Short axis ripple wavelength at the horizontal ice shelf base did not vary measurably in meteoric or basal ice compositions over the 1.5 km transect, which suggests this is a locally appropriate drag coefficient for both meteoric and sediment-laden basal ice.

We can now consider melt rates for both the lower and upper water layers, using mean ocean conditions from ROV and CTD profiles. For the colder, upper layer, with Γ_{TS}=0.006, and S_A = 34.794 g kg^{-1}, Θ = -2.253°C, and ρ_w= 1027.886 kg m^{-3} the melt rate is 0.26 m yr^{-1}. By comparison, the warmer lower layer, with Γ_{TS}=0.006, and S_A = 34.897 g kg^{-1}, Θ = -1.999°C, and ρ_w= 1027.964 kg m^{-3} would drive a hypothetical melt rate of 1.99 m yr^{-1}.

2. (continued) Acknowledgements

We thank the Victoria University of Wellington (VUW) Hot Water Drilling Team led by Darcy Mendeno with Jane Chewings, Hedley Berge, Tim McPhee, Sean Heaphy, Da Gong, and A. Pyne. Logistics support and flights were provided by Antarctica New Zealand (ANZ) and Kenn Borek Air. Additional support was provided by New Zealand National Institute of Water and Atmospheric Research Strategic Science Investment Funding, the Deep South National Science Challenge, and the Antarctic Science Platform. We thank KIS1 chef Lana Hastie and camp manager Jason Watson; Timothy Parkin and Trevor Grigson for camp operations and mechanical support, the equipment traverse team of Bruce Davies, Johno Leitch, Lawrence Kees, and Richie Hunter; and Shulamit Gordon (ANZ) and Elizabeth Kauffman (USAP) for project management. We also acknowledge support from and thank the Polar Geospatial Center for imagery used in field and ROV mission planning. This work benefited from assistance and conversation with other scientists at camp including Peter Dejoux, Arran Whiteford, Martin Forbes, Sara Neuhaus, Andrew Gorman, Matthew Tankersley, Bob Dagg, Jenny Black, Caitlin Hall, and Daniel Price. We additionally thank James Smith for helpful thoughts regarding seafloor geomorphology, and Jacob Buffo and Alex Robel for discussions regarding marine ice formation.

The Aotearoa NZ Ross Ice Shelf Programme was established through an NZARI type B grant specifically to facilitate access to and beneath the Ross Ice Shelf for the national and international scientific community. The hot water drilling system was funded by VUW and managed by the VUW Antarctic Research Centre’s Science Drilling Office. The ASP now carries elements of the original programme forward. Other funding streams, including home

institutions, other NZARI grants, Marsden, and MBIE have contributed to individual projects within this programme.

Supplementary References

68 Robinson, N., Stevens, C. & McPhee, M. Observations of amplified roughness from crystal accretion in the sub-ice ocean boundary layer. *Geophys. Res. Lett.* **44**, 1814-1822 (2017).

69 Holland, D. M. & Jenkins, A. Modeling thermodynamic ice–ocean interactions at the base of an ice shelf. *J. Phys. Oceanogr.* **29**, 1787-1800 (1999).

70 Jenkins, A., Nicholls, K. W. & Corr, H. F. J. Observation and Parameterization of Ablation at the Base of Ronne Ice Shelf, Antarctica. *J. Phys. Oceanogr.* **40**, 2298-2312, doi:10.1175/2010jpo4317.1 (2010).

71 McDougall, T. J. & Wotherspoon, S. J. A simple modification of Newton's method to achieve convergence of order 1+ 2. *Applied Mathematics Letters* **29**, 20-25 (2014).

72 Bushuk, M., Holland, D. M., Stanton, T. P., Stern, A. & Gray, C. Ice scallops: a laboratory investigation of the ice–water interface. *J. Fluid Mech.* **873**, 942-976, doi:10.1017/jfm.2019.398 (2019).

73 Thomas, R. Size of scallops and ripples formed by flowing water. *Nature* **277**, 281-283 (1979).

74 Gwyther, D. E., Galton-Fenzi, B. K., Dinniman, M. S., Roberts, J. L. & Hunter, J. R. The effect of basal friction on melting and freezing in ice shelf–ocean models. *Ocean Modelling* **95**, 38-52 (2015).
